# Induction of aphid resistance in tobacco by the cucumber mosaic virus CMV∆2b mutant is jasmonate‐dependent

**DOI:** 10.1111/mpp.13305

**Published:** 2023-02-12

**Authors:** Warren Arinaitwe, Trisna D. Tungadi, Adrienne E. Pate, Joshua Joyce, Eseul Baek, Alex M. Murphy, John P. Carr

**Affiliations:** ^1^ Department of Plant Sciences University of Cambridge Cambridge UK; ^2^ Present address: Alliance of Bioversity International and International Center for Tropical Agriculture Dong Dok, Ban Nongviengkham, Vientiane Lao People's Democratic Republic; ^3^ Present address: School of Life Sciences, Keele University Newcastle UK; ^4^ Present address: John Innes Centre Norwich UK; ^5^ Present address: Department of Horticultural Sciences Seoul Women's University Seoul Korea

**Keywords:** COI1, green peach aphid, insect vector, nonpersistent transmission, viral suppressor of RNA silencing

## Abstract

Cucumber mosaic virus (CMV) is vectored by aphids, including *Myzus persicae*. Tobacco (*Nicotiana tabacum* ‘Xanthi’) plants infected with a mutant of the Fny strain of CMV (Fny‐CMVΔ2b, which cannot express the CMV 2b protein) exhibit strong resistance against *M. persicae*, which is manifested by decreased survival and reproduction of aphids confined on the plants. Previously, we found that the Fny‐CMV 1a replication protein elicits aphid resistance in plants infected with Fny‐CMVΔ2b, whereas in plants infected with wild‐type Fny‐CMV this is counteracted by the CMV 2b protein, a counterdefence protein that, among other things, inhibits jasmonic acid (JA)‐dependent immune signalling. We noted that in nontransformed cv. Petit Havana SR1 tobacco plants aphid resistance was not induced by Fny‐CMVΔ2b, suggesting that not all tobacco varieties possess the factor(s) with which the 1a protein interacts. To determine if 1a protein‐induced aphid resistance is JA‐dependent in Xanthi tobacco, transgenic plants were made that expressed an RNA silencing construct to diminish expression of the JA co‐receptor CORONATINE‐INSENSITIVE 1. Fny‐CMVΔ2b did not induce resistance to *M. persicae* in these transgenic plants. Thus, aphid resistance induction by the 1a protein requires JA‐dependent defensive signalling, which is countered by the CMV 2b protein.

## Graphical abstract

(a) In tobacco CMV 1a protein stimulates antibiosis against aphids but during infection this is suppressed by the 2b protein through inhibition of jasmonic acid‐regulated gene expression (b) and/or interaction with the 1a protein (c).

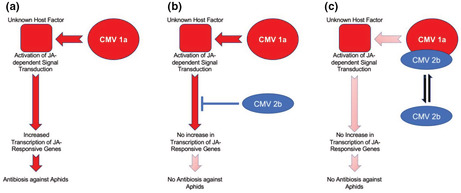

Cucumber mosaic virus (CMV) has a single‐stranded positive‐sense RNA genome that encodes five proteins: the 1a and 2a proteins (encoded by RNAs 1 and 2, respectively), that have primary roles in viral RNA synthesis; the 2b protein (encoded by the second open reading frame of RNA 2), a multifunctional protein that, among other things, counteracts several plant defence mechanisms; and the cell‐to‐cell movement protein and coat protein (encoded respectively by the first and second open reading frames of RNA 3) (Jacquemond, 2012; Palukaitis & García‐Arenal, 2003). CMV infects over 1000 plant species and is vectored in a nonpersistent manner by at least 70 aphid species, including the highly cosmopolitan aphid *Myzus persicae* (Nalam et al., 2019; Yoon et al., 2019). CMV can modify interactions between its host plants and aphid vectors (Carr et al., 2020). For example, when *M. persicae* were caged on plants of the tobacco (*Nicotiana tabacum*) cultivars Xanthi and Xanthi‐nc that were infected with CMV strain Fny (CMV subgroup IA) their survival and reproduction were enhanced, although the rate of aphid growth was decreased (Tungadi et al., 2020; Ziebell et al., 2011). This was seen more consistently on plants infected with LS‐CMV (subgroup II) (Tungadi et al., 2020). Similar positive effects on aphid survival and fecundity were reported for plants of the tobacco line BY4 (Jayasinghe et al., 2021) infected with another subgroup I CMV strain, CMV‐O (Jayasinghe et al., 2022).

However, aphid mortality was drastically increased, and reproduction of surviving aphids was significantly decreased, on tobacco plants infected with Fny‐CMVΔ2b, a mutant of Fny‐CMV that cannot express the 2b protein (Ziebell et al., 2011). In contrast, Tungadi et al. (2020) showed that the corresponding mutant of LS‐CMV (LS‐CMVΔ2b) does not induce resistance to *M. persicae* on tobacco. The factor responsible for eliciting resistance to aphids in tobacco plants infected with Fny‐CMVΔ2b is the Fny‐CMV 1a protein, and it was concluded that during infection by wild‐type Fny‐CMV the resistance‐inducing effect of the Fny‐CMV 1a protein is counteracted by the Fny‐CMV 2b protein (Tungadi et al., 2020). It was also shown that the resistance to *M. persicae* induced by the Fny‐CMV 1a protein could also be counteracted by the 2b protein encoded by LS‐CMV (Tungadi et al., 2020). A different approach used transient silencing of the CMV‐Kurdistan *2b* gene sequence in agroinfiltrated leaf patches of infected Samsun tobacco (Karimi et al., 2022). Here, aphids of *M. persicae*‐*nicotianae* placed on these patches exhibited decreases in performance, supporting a role for the 2b protein in preventing induction of aphid resistance.

Previous work suggested two potential mechanisms by which the Fny‐CMV and LS‐CMV 2b proteins may counteract induction of aphid resistance by the Fny‐CMV 1a protein. First, the triggering of aphid resistance by the 1a protein may be inhibited by a direct interaction with the 2b protein (Watt et al., 2020). However, it should be noted that the direct interaction of the 1a and 2b proteins has been demonstrated only for the Fny‐CMV orthologues. Secondly, aphid resistance induction by the Fny‐CMV 1a protein may depend on the phytohormone jasmonic acid (JA). The 2b proteins of both subgroup IA and subgroup II strains inhibit JA‐regulated gene expression, and so they might inhibit 1a‐induced aphid resistance in this way (Jayasinghe et al., 2022; Lewsey et al., 2010; Westwood et al., 2014). Inhibition of JA‐regulated gene expression by the 2b protein might occur via interaction with JAZ factors (Wu et al., 2017) or through interference with small RNA pathways (Groen et al., 2016; Lewsey et al., 2010), which are known to regulate responses to JA in *Nicotiana* species (Pandey et al., 2008). The two potential mechanisms, that is, interactions between the 1a and 2b proteins (Watt et al., 2020; Westwood et al., 2013) versus inhibition of JA‐dependent signalling by the 2b protein (Lewsey et al., 2010; Westwood et al., 2014) are by no means mutually exclusive. However, in this study we focused on the role of JA‐dependent signalling and gene expression in the induction of aphid resistance by the Fny‐CMV 1a protein expressed in tobacco plants infected with Fny‐CMVΔ2b.

The active form of JA, the JA‐isoleucine conjugate, is perceived by the CORONATINE‐INSENSITIVE 1 (COI1) F‐box protein, which acts via ubiquitination of JASMONATE‐ZIM DOMAIN (JAZ) proteins to regulate, among other things, defence against aphids and other insect herbivores, and floral development (Browse, 2009; Kloth et al., 2016). To investigate the effect of disrupting JA‐dependent defensive signalling on aphid resistance in Fny‐CMVΔ2b infected plants we began experiments using the transgenic *NtCOI1*‐knockdown lines CR3 and CR18, which were developed by Shoji et al. (2008) in the tobacco cultivar Petit Havana SR1. However, we found that in nontransgenic Petit Havana SR1 plants, as well as in the transgenic plants, Fny‐CMVΔ2b induced no resistance against *M. persicae* (Figure S1 and Data S1). This result suggests that whatever factor or factors interact with the 1a protein in Xanthi and Xanthi‐nc (Tungadi et al., 2020; Ziebell et al., 2011) or Samsun tobacco plants (Karimi et al., 2022) are absent or considerably different in properties in Petit Havana SR1.

Therefore, we transformed Xanthi tobacco using *Agrobacterium tumefaciens* (Horsch et al., 1985) carrying the plasmid pBI‐*NtCOI1* to induce knockdown of *NtCOI1* transcript accumulation via RNA silencing (Shoji et al., 2008). *COI1* transcript accumulation was diminished in 12 out of 14 independent tobacco lines carrying the transgene (Table S1 and Figure S2). At the T_0_ generation (primary transformants), the anthers of flowers of plants of four *COI1*‐silenced tobacco lines were observed to be unable to produce pollen grains and could not be used for further work (Figure S3). Male sterility was previously observed by Shoji et al. (2008) with use of the pBI‐*NtCOI1* construct in transgenic tobacco (cvs Petit Havana SR1 and Burley 21) and is consistent with the known role of JA in another development (Browse, 2009). Plants of transgenic lines with decreased *COI1* transcript accumulation but normal floral morphology and fertility were used for further screening. The accumulation of the JA‐regulated *LOX2* transcript is dependent on proper functioning of JA‐dependent defensive signal transduction (Westwood et al., 2014). Changes in *LOX2* induction in response to treatment with methyl‐JA were monitored using an established reverse transcription‐quantitative PCR assay (Westwood et al., 2014) (Figure 1). Four lines of *COI1* knockdown transgenic plants (C1A, C2825LR, Cpb1, and C3A) that were compromised in *LOX2* induction were selected to assess the survival and fecundity of aphids confined on plants that had been infected with Fny‐CMV or Fny‐CMVΔ2b using plants of the T_3_ generation (Figures 2 and S4).

**FIGURE 1:**
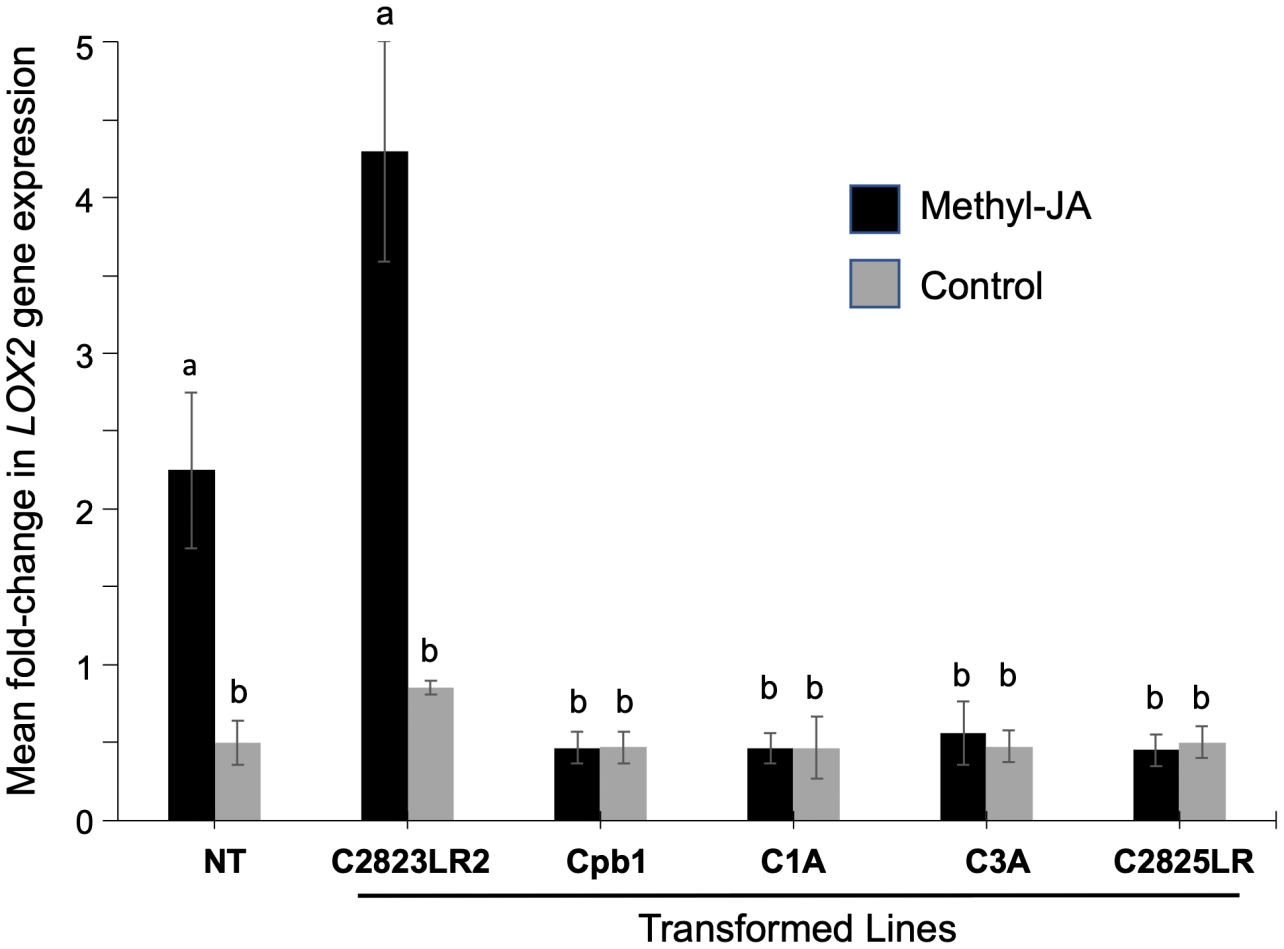
Selection of transgenic tobacco lines with diminished jasmonate‐dependent signalling. Data from screening T_3_ generation cv. Xanthi tobacco plants of lines transformed with pBI‐*NtCOI1* (Shoji et al., 2008) to decrease expression of *CORONATINE‐INSENSITIVE 1* (*COI1*). In this experiment, nontransformed (NT) plants and plants of five transformed lines were sprayed with 250 μM methyljasmonic acid (methyl‐JA) dissolved in 0.05% ethanol or with 0.05% ethanol as a control. Twenty‐four hours later, RNA was extracted from the plants and used for reverse transcription‐quantitative PCR assays to measure the steady‐state accumulation of the *lipoxygenase 2* (*LOX2*) transcript, using the *EF1α* transcript as a control (Westwood et al., 2014). Error bars represent standard error around the mean for three technical replicates. Different lowercase letters indicate statistically significant (α = 0.05) differences in *LOX2* transcript accumulation (analysis of variance and Tukey's post hoc test). Of the transformed lines shown here, only plants of line C2823LR2 failed to show suppression of *LOX2* induction in response to exogenous methyl‐JA.

**FIGURE 2:**
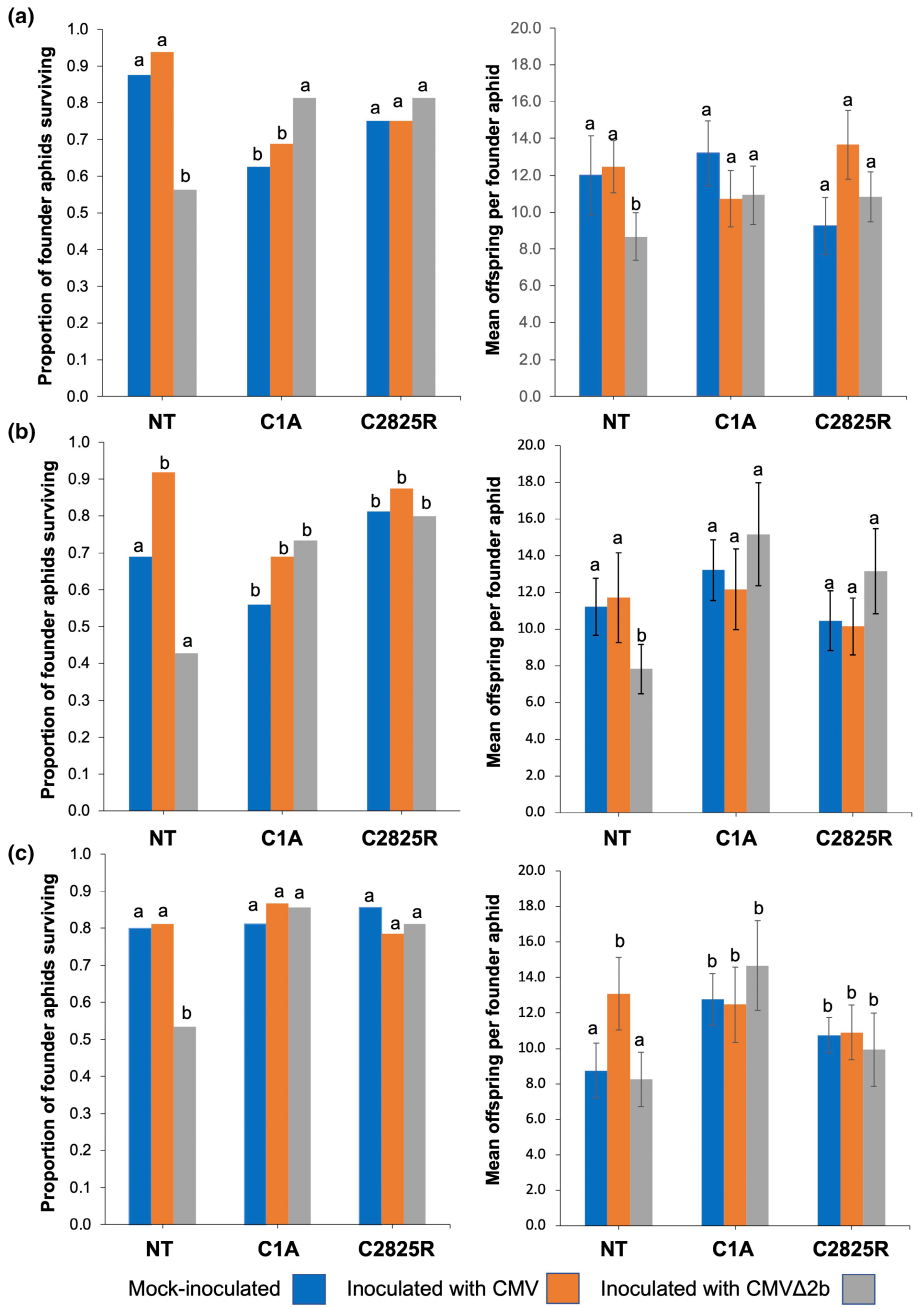
Performance of aphids on plants of tobacco cv. Xanthi. One‐day‐old nymphs of *Myzus persicae* were individually clip‐caged on leaves of plants 10 days following mock inoculation with sterile water or inoculation on lower leaves with virions of wild‐type Fny‐CMV or the mutant, CMVΔ2b. Plants used were nontransformed (NT) plants, or plants belonging to the transgenic lines C1A or C2825R (all in the cv. Xanthi background), which have diminished levels of the *COI1* transcript and decreased responsiveness to exogenous methyl‐JA (Figures 1 and S2). Fourteen days following placement of aphids the survival of the founder aphids (left panels) and the numbers of offspring they produced (right panels) were measured. Both survival and offspring production were recorded in three independent experiments. (a–c) Significant differences in performance, that is, in founder survival and in reproduction were determined using χ^2^ and analysis of variance, respectively. Different lowercase letters denote significant differences (α = 0.05) in aphid performance on infected versus noninfected plants or on transgenic versus nontransgenic plants). Error bars in offspring plots (panels on the right) represent standard error around the mean. For each line the numbers of aphids were (a) mock (*n* = 16), CMVΔ2b (*n* = 16), CMV (*n* = 16); in (b) NT mock (*n* = 13), CMVΔ2b (*n* = 12), CMV (*n* = 12); C1A‐mock (*n* = 16), CMVΔ2b (*n* = 15), CMV (*n* = 16), C2825LR mock (*n* = 16), CMVΔ2b (*n* = 16), CMV (*n* = 16), and in (c) NT mock (*n* = 15), CMVΔ2b (*n* = 15), CMV (*n* = 16); C1A mock (*n* = 16), CMVΔ2b (*n* = 14), CMV (*n* = 15), C2825LR:‐mock (*n* = 16), CMVΔ2b (*n* = 16), CMV (*n* = 14).

Nontransformed and transgenic tobacco plants were grown under controlled conditions and mock‐inoculated with sterile water or inoculated with purified virions of Fny‐CMV or Fny‐CMVΔ2b, and the plants' infection status was confirmed using CMV‐specific double‐sandwich (DAS) ELISA (Bioreba AG), as described previously (Arinaitwe et al., 2022). One‐day‐old nymphs (referred to here as founder aphids) of *M. persicae* (isolate USL1; Devonshire & Sawicki, 1979) were confined individually in clip cages and monitored over the subsequent 14 days for survival and reproduction using previously published methods (Tungadi et al., 2020; Ziebell et al., 2011). Founder aphid survival was diminished on untransformed tobacco plants infected with Fny‐CMVΔ2b compared to mock‐inoculated plants and those infected with Fny‐CMV and colony growth was diminished (Figures 2 and S4), which is in line with previous studies (Tungadi et al., 2020; Ziebell et al., 2011). However, founder aphids caged on plants of the *COI1* knockdown lines C1A and C2825LR (Figure 2), or Cpb1 and C3A (Figure S4) infected with Fny‐CMVΔ2b showed no decrease in survival and no decrease in reproduction. The results, consistent across plants of four independent *COI1* knockdown lines, indicated that decreased reproduction and aphid survival induced by Fny‐CMVΔ2b involve JA‐dependent resistance mechanisms.

The CMV 1a protein interferes with resistance conferred against tobacco mosaic virus (Canto & Palukaitis, 2002) and the 1a protein interacts directly with a range of host factors with effects or potential effects on the CMV infection cycle or on plant immune function. These factors include, among others, the newly characterized transcription factor signalling hub effector 1 (SHE1) (see Yoon & Palukaitis, 2021, and papers referenced therein). Future studies will reveal whether SHE1, or another previously identified interactor, or an unknown factor mediates the triggering by the Fny‐CMV 1a protein of the JA‐dependent aphid resistance. Exploiting tobacco genotype‐specific differences in the ability of the 1a protein to induce resistance to aphids could be valuable in identification of the key interactor(s). Considering the strength of the response, which includes the killing of aphids and a sharp decrease in the fecundity of those aphids that survive, further studies of this system could provide new avenues for controlling these insect pests. For example, by identifying what toxic chemicals are induced in tobacco by infection with Fny‐CMVΔ2b. The most obvious candidate, nicotine, was ruled out in previous work (Ziebell et al., 2011), meaning that novel plant metabolites might be involved. In summary, we have demonstrated that the strong resistance induced in plants infected with Fny‐CMVΔ2b (i.e., by the 1a protein) in Xanthi tobacco plants against *M. persicae* is dependent on the JA‐dependent defensive signal transduction pathway.

## CONFLICT OF INTEREST STATEMENT

The authors declare that they have no conflicts of interest.

## Supporting information


**Figure S1.** Performance of aphids on *COI1* knockdown plants of tobacco cv. Petit Havana SR1.

**Figure S2.** Steady state accumulation of the *NtCOI1* transcript in independently generated transformed Xanthi tobacco lines harboring a *COI1* RNA silencing construct.

**Figure S3.** Some lines of transformed tobacco plants harbouring the *COI1* knockdown construct exhibited male sterility.

**Figure S4.** Performance of aphids on plants of tobacco cv. Xanthi modified to express decreased levels of *COI1*.

**Data S1.** Raw data for aphid performance experiments.

**Table S1.** *COI1* knockdown plant transformation workflow.

## Data Availability

All relevant data are within the paper and its Supporting Information files.
