# Supplementary material for: Induction of aphid resistance in tobacco by the cucumber mosaic virus CMV∆2b mutant is jasmonate‐dependent
Source: Mol Plant Pathol. 2023 Feb 12;24(4):391–5. doi: 10.1111/mpp.13305 (PMC10013749; doi:10.1111/mpp.13305)
Supplement: Supplementary file 1 — Figure S1. Performance of aphids on COI1 knockdown plants of tobacco cv. Petit Havana SR1. [file MPP-24-391-s005.pdf]

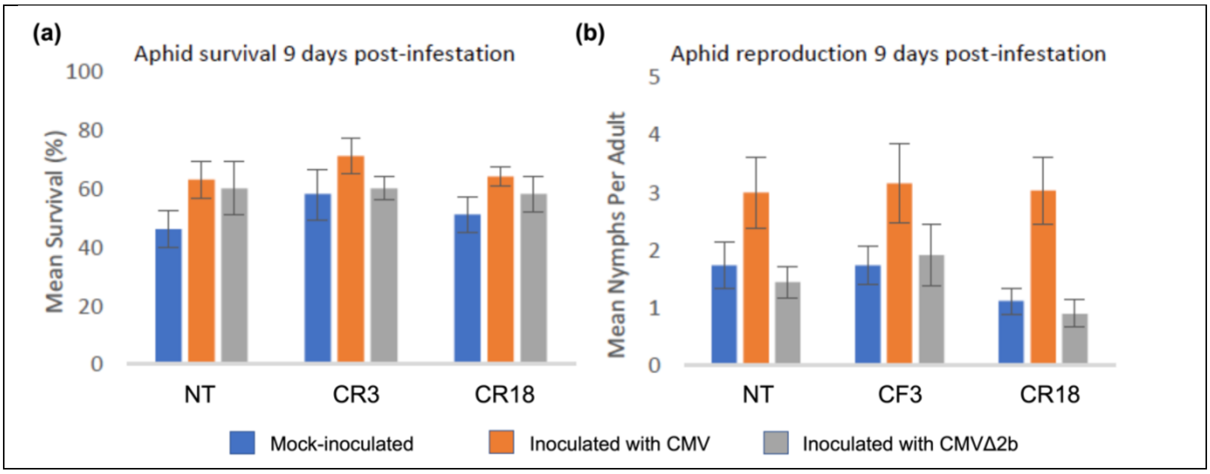

Figure S1. Performance of aphids on *COI1* knockdown plants of tobacco cv. Petit Havana SR1. One day old nymphs of *Myzus persicae* were individually clip-caged on leaves of plants 10 days following mock inoculation with sterile water or inoculation on lower leaves with virions of wild-type Fny-CMV or of the CMVΔ2b mutant. Plants used were non-transformed (NT) Petit Havana SR1, or plants belonging to the transgenic lines CR3 or CR18 (both in the Petit Havana SR1 background), which have diminished levels of the *COI1* transcript and have been described previously by Shoji and colleagues (2008). Nine days following placement of aphids the survival of the founder aphids **(a)** and the progeny they produced **(b)** counted. Error bars represent standard error around the mean. There were no statistically significant (α= 0.05: Tukey's HSD post hoc test) differences in aphid performance on infected versus non-infected plants or on transgenic versus non-transgenic plants.
