# Supplementary material for: Induction of aphid resistance in tobacco by the cucumber mosaic virus CMV∆2b mutant is jasmonate‐dependent
Source: Mol Plant Pathol. 2023 Feb 12;24(4):391–5. doi: 10.1111/mpp.13305 (PMC10013749; doi:10.1111/mpp.13305)
Supplement: Supplementary file 2 — Figure S2. Steady state accumulation of the NtCOI1 transcript in independently generated transformed Xanthi tobacco lines harboring a COI1 RNA silencing construct. [file MPP-24-391-s006.pdf]

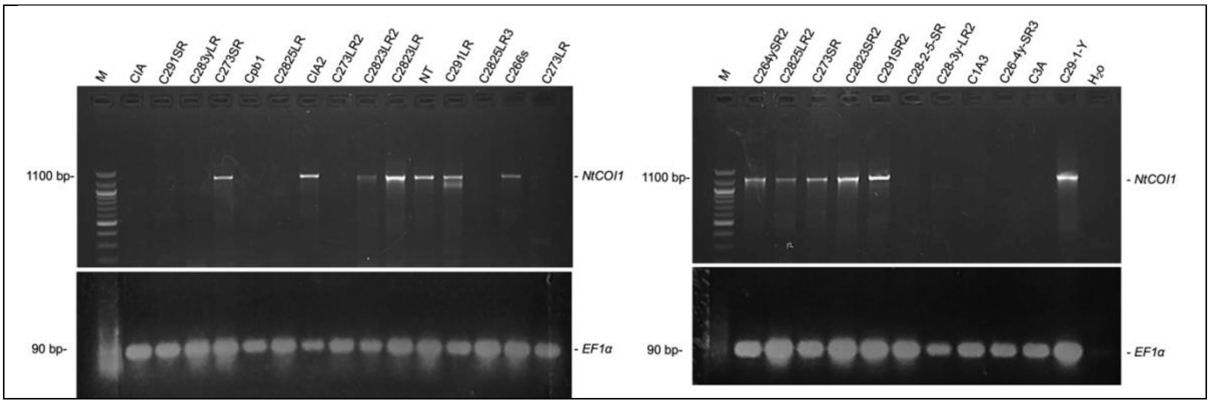

Figure S2. Steady state accumulation of the *NtCOI1* transcript in independently generated transformed Xanthi tobacco lines harboring a *COI1* RNA silencing construct. RT-PCR was performed on RNA extracted from leaves of non-transformed (NT) tobacco plants and T_0 generation plants of the lines indicated. More information on these tobacco lines appears in Table S1. The degenerate primer pair YTIAAYTAYATGACIGA and GCICKYTCISWRAARCARCA was used for detection of the *NtCOI1* transcript (in which, Y = C or T, I = Inosine, R = A or G, K = G or T, and W = A or T). The previously described GCATGCGTCAAACTGTTGCTGT and TTCTTCTGAGCAGCCTTGGTGA primer pair was used to amplify the *EF1α* transcript as an internal control (Westwood et al., 2014). The PCR amplicons of *NtCOI1* and *EF1α* and their sizes in base pairs (bp) are indicated alongside DNA markers (lanes M). H_2O indicates a control PCR reaction lacking cDNA template. Lines showing no detectable accumulation of the *NtCOI1* transcript were further characterized for responsiveness to jasmonate treatment (e.g., Figure 1).
