# Supplementary material for: Induction of aphid resistance in tobacco by the cucumber mosaic virus CMV∆2b mutant is jasmonate‐dependent
Source: Mol Plant Pathol. 2023 Feb 12;24(4):391–5. doi: 10.1111/mpp.13305 (PMC10013749; doi:10.1111/mpp.13305)
Supplement: Supplementary file 3 — Figure S3. Some lines of transformed tobacco plants harbouring the COI1 knockdown construct exhibited male sterility. [file MPP-24-391-s001.pdf]

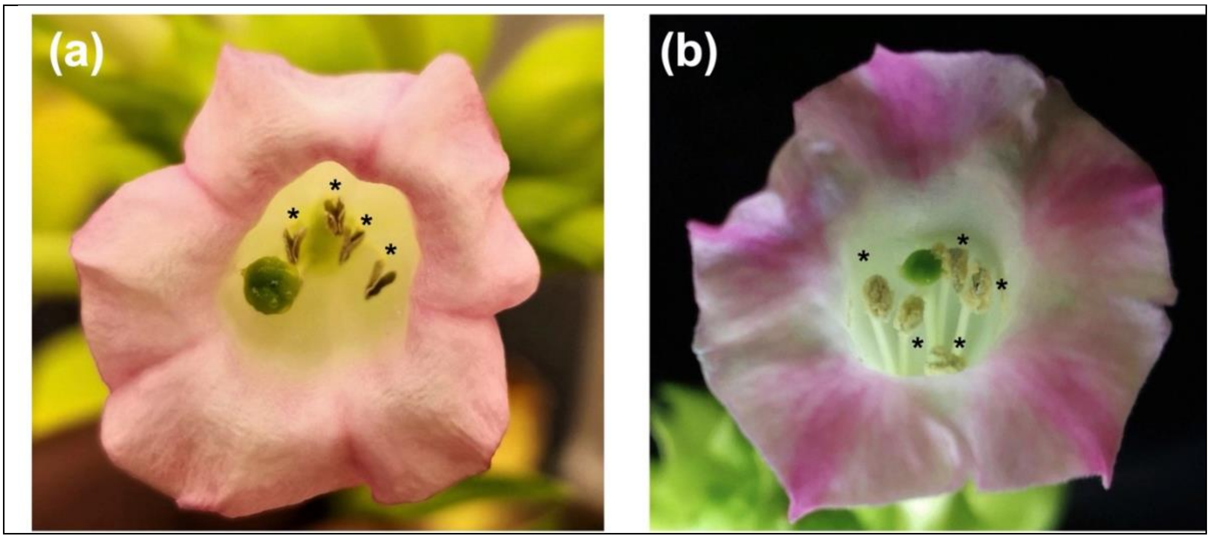

Figure S3. Some lines of transformed tobacco plants harbouring the *COI1* knockdown construct exhibited male sterility. Panel (a) shows the appearance of anthers (*) in a flower produced by the T_0 generation transformed plant of line C273LR2 which, in contrast to the anthers of a non-transformed tobacco plant (cv. Xanthi) (b), do not release pollen grains. Plants were photographed 5 days after bud appearance. This male sterility was also observed in flowers of the T_0 plants of lines C291SR, C283yLR, and C273LR (Table S1).
