# Supplementary material for: Induction of aphid resistance in tobacco by the cucumber mosaic virus CMV∆2b mutant is jasmonate‐dependent
Source: Mol Plant Pathol. 2023 Feb 12;24(4):391–5. doi: 10.1111/mpp.13305 (PMC10013749; doi:10.1111/mpp.13305)
Supplement: Supplementary file 4 — Figure S4. Performance of aphids on plants of tobacco cv. Xanthi modified to express decreased levels of COI1. [file MPP-24-391-s003.pdf]

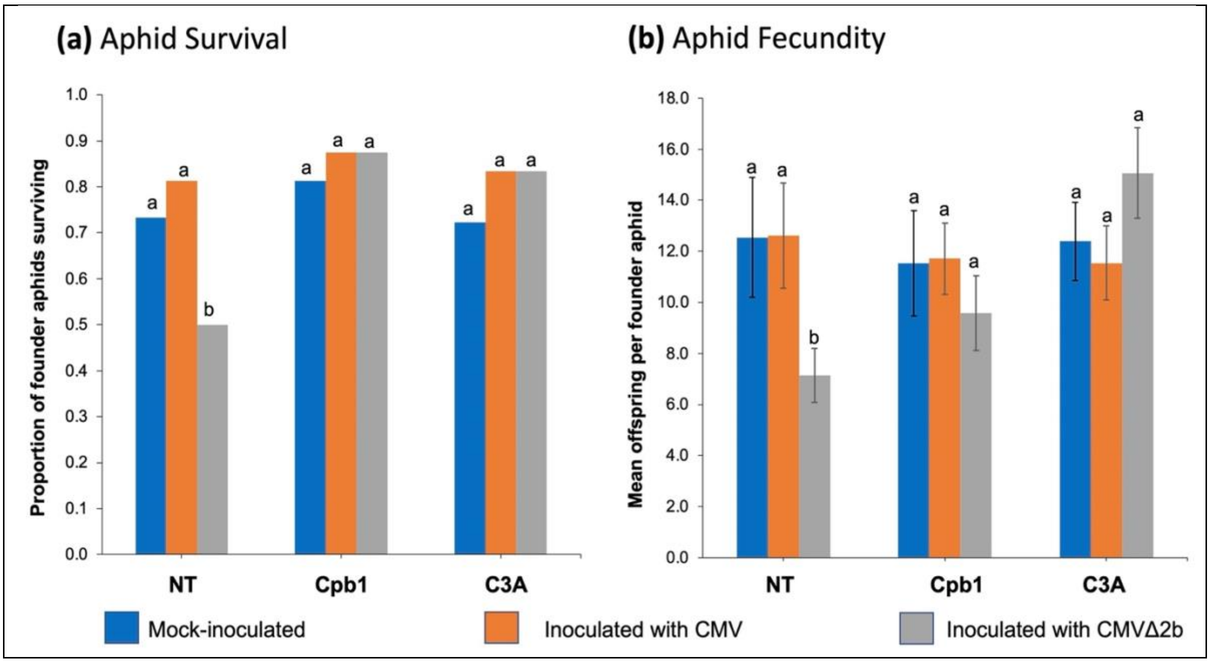

Figure S4. Performance of aphids on plants of tobacco cv. Xanthi modified to express decreased levels of COI1. One day old nymphs of *Myzus persicae* were individually clip-caged on leaves of plants 10 days following mock inoculation with sterile water or inoculation on lower leaves with virions of wild-type Fny-CMV or the mutant, CMVΔ2b. Plants used were non-transformed (NT) Xanthi, or plants belonging to the transgenic lines Cpb1 or C3A (both in the Xanthi background), which have diminished levels of the *COI1* transcript and decreased responsiveness to jasmonate treatment (Figure 1, Figure S2). Fourteen days following placement of aphids the survival of the founder aphids (a) and the numbers of progeny they produced (b) were measured. The difference in performance i.e., survival and progeny size were determined using Chi-square and ANOVA, respectively. Different letters denote statistically significant differences (α= 0.05) in aphid performance on infected versus non-infected plants or on transgenic versus non-transgenic plants determined using Tukey's HSD *post hoc* test. Error bars in (b) represent standard error around the mean.
