# Supplementary material for: Induction of aphid resistance in tobacco by the cucumber mosaic virus CMV∆2b mutant is jasmonate‐dependent
Source: Mol Plant Pathol. 2023 Feb 12;24(4):391–5. doi: 10.1111/mpp.13305 (PMC10013749; doi:10.1111/mpp.13305)
Supplement: Supplementary file 6 — Table S1. COI1 knockdown plant transformation workflow. [file MPP-24-391-s004.pdf]

Table S1. *COI1* knockdown plant transformation workflow. The table indicates phenotypic and molecular differences between plants of transformed lines and non-transformed (NT) tobacco plants. Tobacco lines in grey rows were selected for aphid experiments.

Line ID	Kanamycin resistance confirmed	T-DNA detected at T_0 generation[1]	Knockdown of *COI1* expression[2]	Pollen production	Effects on JA-responsive gene expression[3]
**C1A**	✓	✓	✓	**Normal**	**Reduced**
C291SR	✓	✓	✓	Impaired	Not tested
C283yLR	✓	✓	✓	Impaired	Not tested
C273SR	✓	×			
**Cpb1**	✓	✓	✓	**Normal**	**Reduced**
**C2825LR**	✓	✓	✓	**Normal**	**Reduced**
C1A2	✓	×		Normal	Not tested
C273LR2	✓	✓	✓	Impaired	Not tested
C2823LR2	✓	✓	✓	Normal	No change
C2823LR	✓	×			
C291LR	✓	×			
C2825LR3	✓	✓	✓	Normal	Not tested
C266s	✓	×			
C273LR	✓	✓	✓	Impaired	Not tested
C264ySR2	✓	×			
C2825LR2	✓	×			
C273SR	✓	×			
C2823SR2	✓	×			
C291SR2	✓	×			
28-2-5-SR	✓	✓	✓	Normal	Not tested
28-3y-LR2	✓	✓	✓	Normal	Not tested
C1A3	✓	✓	✓	Normal	Not tested
**C3A**	✓	✓	✓	**Normal**	**Reduced**
26-4y-SR3	✓	✓	✓	Normal	Not tested

Notes

[1] Confirmed by PCR. Check marks indicate successful detection of the T-DNA using PCR and crosses indicate that no PCR amplicon was generated.

[2] Decreased *COI1* transcript accumulation confirmed by RT-PCR at T_0 generation (Figure S2)

[3] Transcript accumulation of *NtLOX2* measured by RT-qPCR (Figure 1)
